# Computed Tomography Coronary Angiography and Computational Fluid Dynamics Based Fractional Flow Reserve Before and After Percutaneous Coronary Intervention

**DOI:** 10.3389/fbioe.2021.739667

**Published:** 2021-09-07

**Authors:** Gaurav Chandola, Jun-Mei Zhang, Ru-San Tan, Ping Chai, Lynette Teo, John C. Allen, Ris Low, Weimin Huang, Shuang Leng, Jiang Ming Fam, Chee Yang Chin, Ghassan S. Kassab, Adrian Fatt Hoe Low, Swee Yaw Tan, Terrance Chua, Soo Teik Lim, Liang Zhong

**Affiliations:** ^1^ National Heart Centre Singapore, Singapore, Singapore; ^2^ Duke-NUS Medical School, Singapore, Singapore; ^3^ Department of Cardiology, National University Heart Centre, Singapore, Singapore; ^4^ Yong Loo Lin School of Medicine, National University of Singapore, Singapore, Singapore; ^5^ Department of Diagnostic Imaging, National University Hospital, Singapore, Singapore; ^6^ Institute for Infocomm Research, Agency for Science, Technology and Research, Singapore, Singapore; ^7^ California Medical Innovations Institute, San Diego, CA, United States

**Keywords:** fractional flow reserve, stents, hemodynamics, coronary angiography, computed tomography angiography

## Abstract

Invasive fractional flow reserve (FFR) is recommended to guide stent deployment. We previously introduced a non-invasive FFR calculation (FFR_B_) based on computed tomography coronary angiography (CTCA) with reduced-order computational fluid dynamics (CFD) and resistance boundary conditions. Current study aimed to assess the feasibility and accuracy of FFR_B_ for predicting coronary hemodynamics before and after stenting, with invasive FFR as the reference. Twenty-five patients who had undergone CTCA were prospectively enrolled before invasive coronary angiography (ICA) and FFR-guided percutaneous coronary intervention (PCI) on 30 coronary vessels. Using reduced-order CFD with novel boundary conditions on three-dimensional (3D) patient-specific anatomic models reconstructed from CTCA, we calculated FFR_B_ before and after virtual stenting. The latter simulated PCI by clipping stenotic segments from the 3D coronary models and replacing them with segments to mimic the deployed coronary stents. Pre- and post-virtual stenting FFR_B_ were compared with FFR measured pre- and post-PCI by investigators blinded to FFR_B_ results. Among 30 coronary lesions, pre-stenting FFR_B_ (mean 0.69 ± 0.12) and FFR (mean 0.67 ± 0.13) exhibited good correlation (*r* = 0.86, *p* < 0.001) and agreement [mean difference 0.024, 95% limits of agreement (LoA): −0.11, 0.15]. Similarly, post-stenting FFR_B_ (mean 0.84 ± 0.10) and FFR (mean 0.86 ± 0.08) exhibited fair correlation (*r* = 0.50, *p* < 0.001) and good agreement (mean difference 0.024, 95% LoA: −0.20, 0.16). The accuracy of FFR_B_ for identifying post-stenting ischemic lesions (FFR ≤ 0.8) (residual ischemia) was 87% (sensitivity 80%, specificity 88%). Our novel FFR_B_, based on CTCA with reduced-order CFD and resistance boundary conditions, accurately predicts the hemodynamic effects of stenting which may serve as a tool in PCI planning.

## Introduction

Revascularization of coronary artery ischemic lesions with stents by percutaneous coronary intervention (PCI) has proven to be an effective therapeutic method for patients with symptomatic coronary artery disease (CAD) (Erne et al., 2007; Shaw et al., 2008). Significant residual ischemia may occur, however, in approximately 5–24% of patients after stenting (Pijls et al., 2000; Jeremias et al., 2019). Therefore, a tool to predict the hemodynamic effect of revascularization is vital to enable appropriate stenting strategies.

Fractional flow reserve (FFR) is the gold standard for assessing the hemodynamic significance of stenosis (Tonino et al., 2009). Use of FFR to guide PCI enables improved clinical and economic outcomes of stenting (Tonino et al., 2009; Fearon et al., 2010). Given the invasive and time-consuming nature of FFR, its adoption as clinical routine has been somewhat less enthusiastic than anticipated (Zhang D. et al., 2015). An added complication involves measurement of a side branch after stenting, which is challenging because the jailed side branch must be re-crossed through the stent strut using a pressure wire/catheter.

Computational fluid dynamics (CFD) methods have been combined with medical images to derive non-invasive FFR (Min et al., 2012; Nørgaard et al., 2014; Zhang et al., 2016; Zhang et al., 2018; Zhong et al., 2018) and develop virtual stenting approaches to assist PCI treatment planning (Kim et al., 2014; Gosling et al., 2019). Although these CFD based approaches have shown a high level of accuracy when compared to invasive FFR measurements (Koo et al., 2011; Min et al., 2012; Taylor et al., 2013; Nørgaard et al., 2014), the demand on computational time is significant [1–4 h (Nørgaard et al., 2014) or 6 h (Koo et al., 2011) per simulation] due to the transient simulations. This limits the utility of these methods in routine clinical practice where real-time results are needed. Attempts to reduce computational time have employed reduced order methods whereby vessels are modeled as 1D segments in CFD simulations. Although this results in a significant reduction in computational time (5–10 min per simulation), only fair correlation (e.g., Pearson correlation of 0.59) with invasive FFR is achieved (Coenen et al., 2015). Some CFD schemes use generic outlet boundary conditions in the absence of patient-specific data at the distal model boundaries (Gosling et al., 2019), but do not attain the level of accuracy obtained by applying personalized distal boundary conditions.

We previously introduced a reduced-order CFD method and novel boundary conditions (FFR_B_) for obtaining FFR non-invasively from computed tomography coronary angiography (CTCA) images based on steady state flow simulations (Zhang et al., 2016). Here “B” represents “Bernoulli”, as it signifies the importance of fluid mechanics in the non-invasive FFR calculation. It requires less computational time and has demonstrated excellent accuracy compared with invasive FFR measurements (Zhang et al., 2016; Zhang et al., 2018; Zhang et al., 2020). The goal of this study is to assess the performance of FFR_B_ in predicting coronary hemodynamics before and after stenting, with invasive FFR as the reference.

## Materials and Methods

### Study Design and Population

The study was carried out at the National Heart Centre Singapore and National University Hospital Singapore. Participants of this study were recruited from an ongoing observational cohort study that aimed to compare the diagnostic performance of FFR_B_ against invasive FFR in patients with intermediate coronary artery disease (ClinicalTrials.gov Identifier: NCT03054324). Consecutive patients attending two tertiary cardiology centers aged 21–98 years who had undergone CTCA and were scheduled to undergo clinically indicated coronary angiography (with invasive FFR performed to vessels having diameter stenoses between 30 and 90%) within 180 days were eligible to participate. The inclusion criteria of the trial were as follows: 1) Suspected or known CAD patients aged ≥21 years; and 2) Patients had undergone CTCA within 6 months and were scheduled to undergo clinically indicated coronary angiography with FFR measurement. Exclusion criteria were 1) Prior PCI; 2) Cardiac event and/or coronary revascularization between CTCA and invasive coronary angiography (ICA) (Bashore et al., 2012); 3) Angina at rest; 4) Left ventricular ejection fraction <30%; 5) Hypertrophic cardiomyopathy, significant valve disease including prosthetic heart valve, implanted pacemaker or defibrillator, complex congenital heart disease; 6) Estimated glomerular filtration rate <30 ml/min/1.73 m^2^; 7) Tachycardia or significant arrhythmia; 8) iodinated contrast allergy; 9) Contraindication to beta blocker, nitroglycerin or adenosine; 10) Atrial fibrillation; and 11) Serious comorbidity with life expectancy <2 years and pregnancy. The local institutional review boards approved the protocol for this clinical trial. All recruited subjects gave written informed consent. From September 20, 2016 to March 25, 2020, 117 participants were recruited for the trial.

As a sub-study of the multicenter prospective trial, this study consecutively enrolled a total of 27 patients who had undergone elective stenting with FFR measurements before and after stenting in at least one vessel. As the image quality in two patients was inadequate to allow satisfactory segmentation, a total of 25 patients were included in the final analysis.

### CTCA Acquisition and Image Segmentation

Following SCCT guidelines (Abbara et al., 2016), CTCA was performed on the following CT scanners: Toshiba Aquilion One 320 slice, Canon Aquilion ONE Genesis 640 Slice, Siemens Somatom Force dual source 384-dector and Philips Brilliance iCT 256-detector. Detailed scanner information is summarized in Supplementary Table 1. Beta-blockers were administrated to patients with resting heart rate >65 beats/minute, and sublingual glyceryl trinitrate was administered prior to each scan. Prospective ECG-triggered scanning mode was used for all CTCA scans.

All acquired CTCA images were processed and reconstructed into 3D patient-specific coronary models. The images were initially classified as diagnostic or non-diagnostic following independent review by two experienced imaging specialists blinded to the analyses. Severe motion artefacts, stair-step artefacts and image noise were the main reasons for non-diagnostic CTCA images. Coronary segmentation was performed on segments with diameter ≥1.5 mm using semi-automated software (QAngio CT, Research Edition v3.0.37.0, Medis) (Collet et al., 2018). Segmentation was performed as coronary arteries were divided into 17 segments based on the American Heart Association recommendation (Austen et al., 1975). Centerline of the coronary artery was first obtained using a fast vessel tracking algorithm and a stretched multi planar-reformatted (MPR) volume was created accordingly. Second, four longitudinal cross sections were extracted from the MPR volume at 45° angular intervals. Lumen contours in these longitudinal images were detected with a model-guided minimum cost approach (MCA) (Van der Zwet et al., 1989). Third, the lumen contours were detected in each transverse slice of the MPR volume using MCA with a circular lumen model. The intersection points of each transverse slice with the longitudinal contours aided the contour depiction.

The segmented contours of each vessel were subsequently imported into 3D Workbench (Medis) to generate the surface meshes and facilitate reconstruction of a 3D model in ANSYS SpaceClaim (Supplementary Figure 1).

### ICA and FFR Measurement

ICA was performed in accordance with standard clinical guidelines (Bashore et al., 2012). The decision to perform invasive FFR measurements was made at the discretion of the interventionalist based on severity of the stenosis, lesion characteristics and clinical experience. Of note, interventionalists at the two participating hospitals were encouraged to perform invasive FFR for intermediate coronary stenosis to guide revascularization decisions in accordance with guidelines, as well as after coronary stenting. PCI were performed by the interventionalists based on ICA, invasive FFR and clinical considerations, as applicable. FFR measurements were carried out using one of the following devices: PressureWire Aeris guidewire (St Jude Medical), ACIST Navvus microcatheter (Medtronic), Volcano Verrata pressure guidewire (Philips), or Certus PressureWireX guidewire (Abbott). The intra-coronary pressure was measured in at least one vessel following either intravenous infusion (140–180 μg/kg/min) or intracoronary bolus (60–200 µg) of adenosine to induce hyperaemia (Supplementary Figure 2). Systolic (SBP) and diastolic brachial blood pressure (DBP) measurements were carried out before the ICA examination. Mean blood pressure (MBP) was calculated using Eq. 1 and used in the calculation of non-invasive FFR_B._

MBP=DBP+(SBP−DBP)/3 (1)

Lesions with invasive FFR ≤0.80 were considered hemodynamically significant (Pijls et al., 1996). Lesions were also classified as focal, bifurcation, ostial and tandem lesions. Focal lesions were single isolated lesions present in a vessel path from ostium to the distal segment; bifurcation lesions extended across a branch; ostial lesions occurred at the junction of the aorta and coronary artery or the take-off of the daughter branch artery from the parent vessel. Tandem lesions had two or more lesions in a vessel path with ≥50% diameter stenosis (Supplementary Figure 3).

### FFR_B_ Determination and Virtual Stenting

**FIGURE 1:**
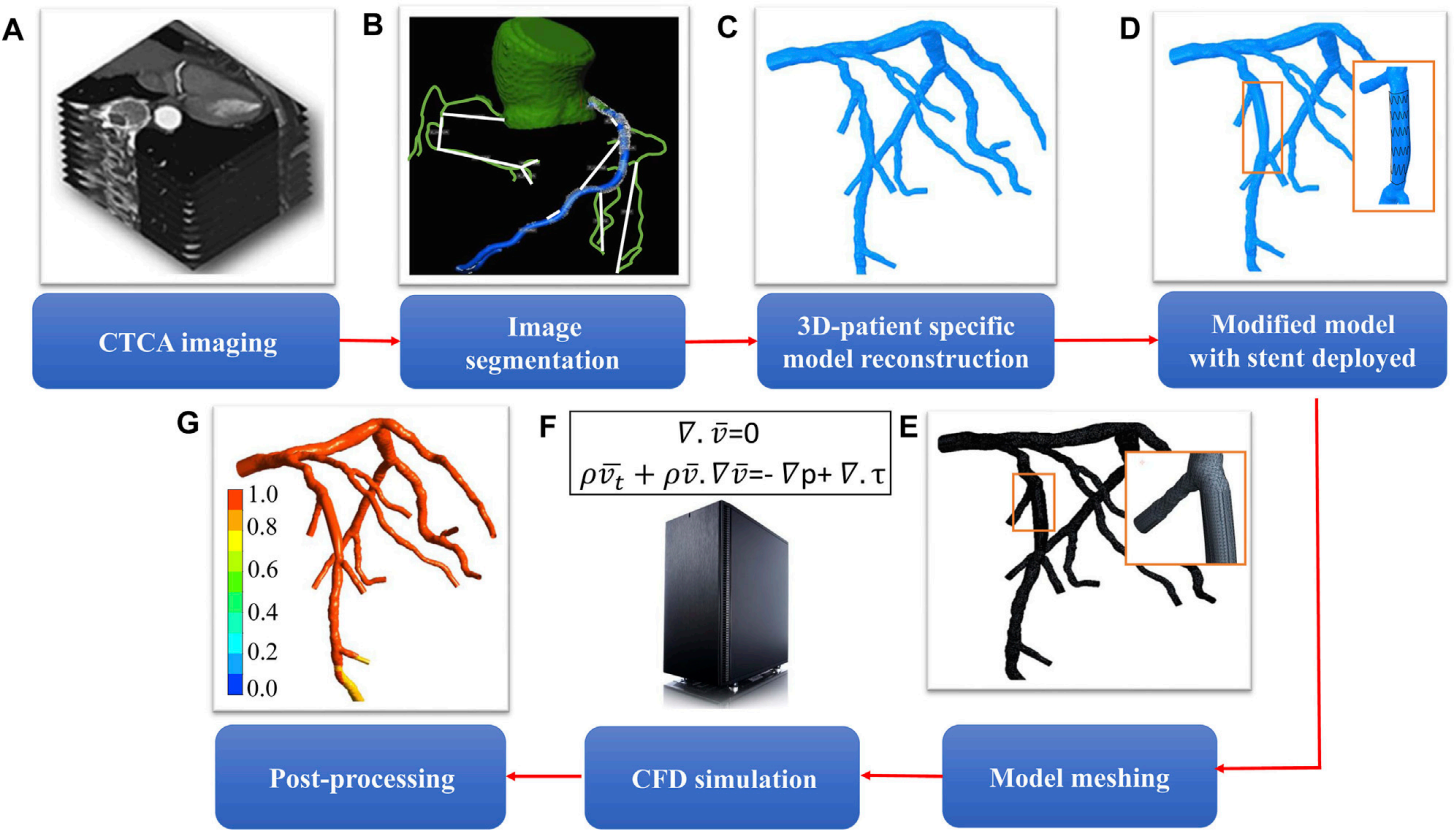
Workflow for determining the hemodynamic effect of revascularization using virtual stenting and noninvasive FFR_B_ **(A)** acquisition of CTCA images **(B)** segmentation of acquired images **(C)** 3D reconstruction of patient-specific coronary artery tree **(D)** model modification to replace the stenotic region with a virtual stent of desired geometric characteristics **(E)** mesh generation to discretize the 3D model **(F)** solution of mass and momentum conservation equations to simulate blood flow in coronary arteries **(G)** post-processing to obtain non-invasive FFR_B_.

The workflow to compute FFR_B_ is shown in Figure 1 and described in our previous study (Zhang et al., 2016). In short, the 3D model was imported into ANSYS Meshing (ANSYS) to discretize the computational domain into tetrahedral elements with boundary inflation layers, the total cell count ranging from 0.3 to 0.8 million cells. Subsequently, FLUENT (ANSYS) was used to solve the continuity and Navier-Stokes equations. Blood was modeled as a Newtonian fluid. The inlet static pressure was set at 6.8 mmHg lower than the patient-specific MBP in consideration of the hyperemia effect of adenosine (Wilson et al., 1990). Resistance boundary conditions were specified at each outlet boundary of the model to mimic physiological conditions. At each coronary outlet, the pressure was related to the reference pressure (*P*_0_) and the resistance of downstream vasculature of each coronary branch (*R*_i_). They were updated with an under-relaxation scheme during iterations until the total outflow from all the outlets matched the inflow rate at hyperemia. The detailed derivation of the formulations for the outlet boundary condition can be found in the Supplementary Material. User-defined functions modeling the coronary microvasculature were used to mimic the outlet physiology. It comprises algorithm to implement the resistance boundary conditions at the outlet of the model, based on a novel iterative scheme to determine the resistance and reference pressure of each coronary vessel (Zhang et al., 2016). The CFD simulation relied on several physiological assumptions (Koo et al., 2011; Nørgaard et al., 2014). First, the resting total coronary blood flow rate was related to myocardial mass measured from patient-specific CTCA (Hamada et al., 1998; Wieneke et al., 2005). Second, the resistance of each coronary branch at rest was assumed to be proportional to the size of the parent and daughter branch vessels (Zhou et al., 1999). Third, coronary resistance was taken to be 0.21 times the resting value to mimic hyperemia (Wilson et al., 1990). A no-slip boundary condition was set for the wall. FFR_B_ ≤0.8 was deemed as indicating ischemia. To ensure identical locations of FFR_B_ and invasive FFR measurements, two interventionists blinded to non-invasive FFR_B_ results visually reviewed the ICA images and then marked the FFR measurement locations on the 3D models reconstructed from CTCA images. The values of the invasive FFR measured across coronary artery stenoses before and after stenting served as ground truths against which we compare the corresponding CFD-derived FFR_B_ values in the pre-stenting CTCA models as well as in the simulated post-stenting CTCA models with coronary anatomy reconstituted using actual implanted stents’ geometries and locations.

**FIGURE 2:**
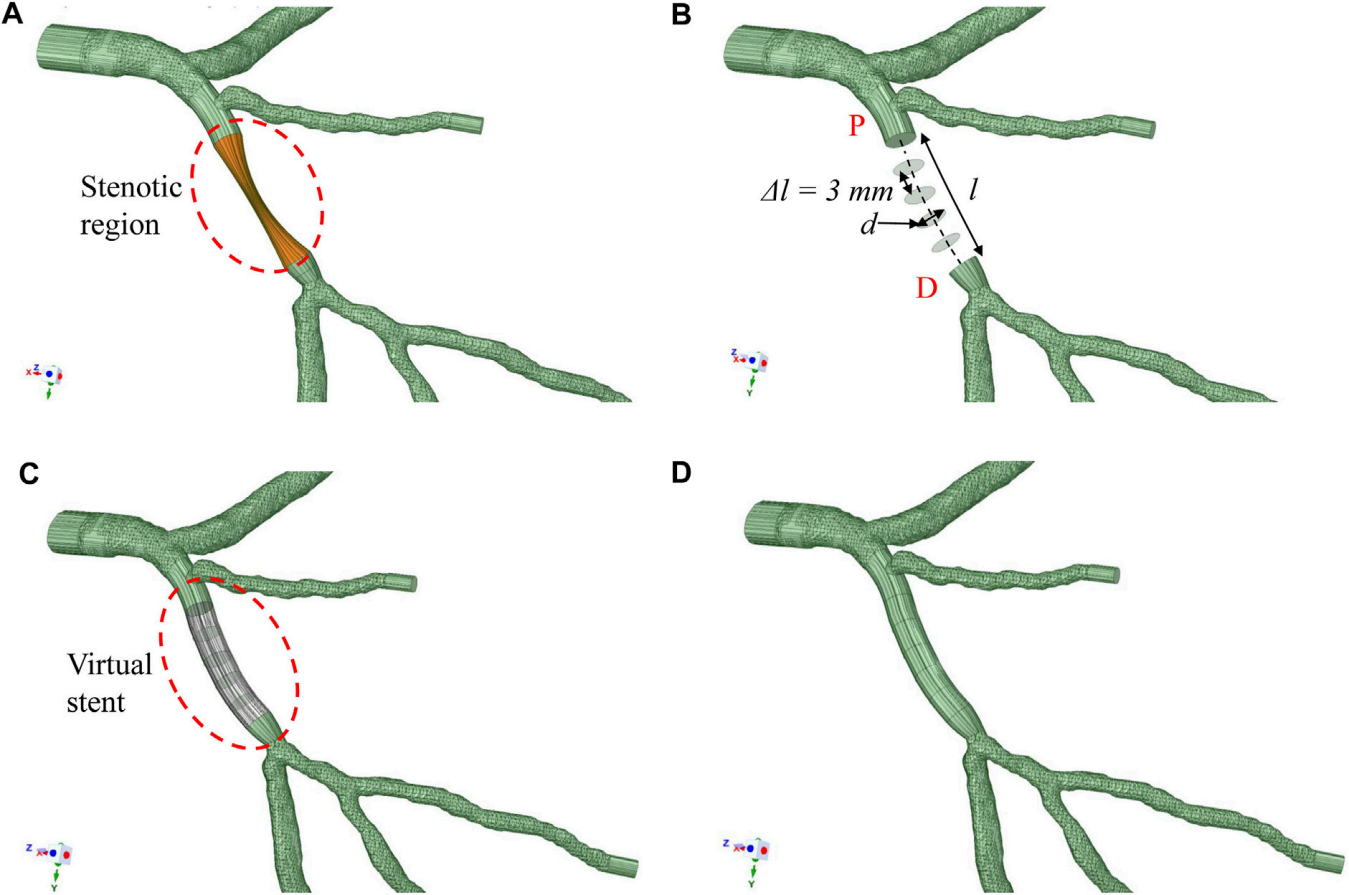
Methodology to deploy stent **(A)** patient-specific 3D model with the stenotic region highlighted **(B)** The diseased section is clipped between the proximal (P*) and distal (D*) locations. A series of circular surfaces are generated along and perpendicular to the original lumen centerline. These circular surfaces have diameters equal to the virtual stent and spaced at 3 mm interval apart from one another **(C)** These circular surfaces and original surfaces at “P*” and “D*” are interpolated with B-spline interpolation to form a virtual stent segment **(D)** The virtual stent segment is merged with the rest of the segments in the original model to form a new model to mimic the post-stenting scenario.

**FIGURE 3:**
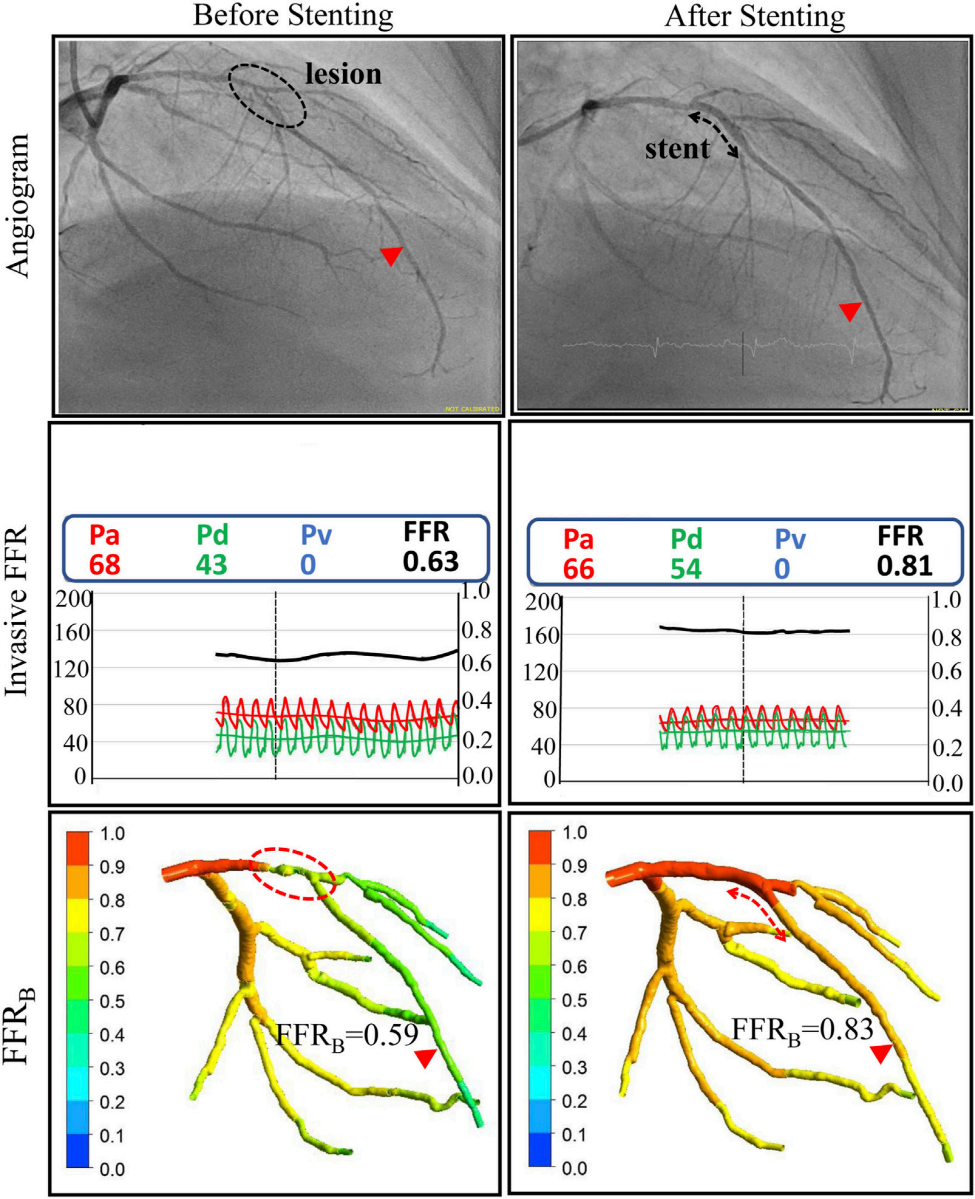
Comparison of invasive and non-invasive functional assessment before and after coronary stenting for one of the patients. Left column: ICA and invasive FFR confirmed the presence of a functionally significant lesion on the Left Anterior Descending (LAD) coronary artery with an FFR value of 0.63. Non-invasive FFR (FFR_B_) obtained using CTCA imaging successfully classified the lesion as functionally significant with an FFR_B_ value of 0.59. Right Column: After stent implantation invasive FFR was 0.81 and the corresponding FFR_B_ calculated on virtual stenting was 0.83, both indicating no residual ischemia.

ANSYS SpaceClaim (ANSYS) was used to mimic the 3D model with deployed stent. The stenotic segment was clipped and replaced by a segment with appropriate diameter and length to replicate the dimensions of the deployed stent(s) during PCI. The reconstructed segment was constructed by B-spline interpolation of a set of circular cross-sections, which followed the lumen centerline with diameters identical to those of the stent (Figures 2B,C). The stent deployment location was inferred from the angiograms and checked by two experienced interventionists to facilitate the comparison between FFR_B_ and FFR post-stenting. Figures 2A–D show the procedures used to generate the model with virtual stenting and FFR_B_ was subsequently computed. This procedure can be generalized to accommodate a stent of any diameter and length in testing the effects of different stenting strategies. Examples of FFR_B_ at locations corresponding to invasive FFR measurements are shown in Figure 3.

### Statistical Analysis

Continuous and categorical variables were reported as mean ± standard deviation (SD), and frequencies and/or percentages, respectively. The correlations between FFR_B_ and invasive FFR before and after stenting were assessed using Pearson correlation. Bland-Altman plots were used to determine the agreement between FFR_B_ and invasive FFR. Diagnostic accuracy of FFR_B_ with invasive FFR as the reference was assessed by calculating sensitivity, specificity, positive and negative predictive values, and raw accuracy. All statistical analyses were performed using SPSS version 22 (IBM Inc., Armonk, NY, United State). Statistical significance was set at *p* ≤ 0.05.

### Intra and Inter Observer Reproducibility

To evaluate reproducibility and observer concordance of FFR_B_ measurement, intra-observer and inter-observer variability were studied on a randomly selected 10 cases before and after stenting using Bland-Altman analysis, intra-class correlation (ICC) and coefficient of variation (COV). In assessing inter-observer variability, measurements were repeated by a second independent observer (Author ZJM) blinded to the first observer’s (Author GC) results. To address intra-observer variability, repeated analyses 37 days apart were performed by the same observer on the same 10 cases.

## Results

### Baseline Clinical and Lesion Characteristic Variables

ICA and FFR measurements were performed 22 (interquartile range 14–32) days after CTCA scan for 25 patients. Baseline clinical and lesion characteristic variables are summarized in Table 1. Mean age was 59 ± 9 years; 21 (84%) patients were male; 8 (32%) patients had diabetes mellitus. The mean calcium score was 349. The number of focal, bifurcation, ostial and tandem lesions was 12, 10, 3 and 5, respectively. In total, 30 vessels (22 left anterior descending (LAD), four left circumflex (LCX), and four right coronary arteries (RCA)) were treated with 36 drug eluting stents (DES). The mean length and diameter of the stent was 26 ± 11 mm and 2.96 ± 0.50 mm respectively (Table 2). The histogram of invasive FFR values before and after stenting is shown in Supplementary Figure 4.

**TABLE 1:** Baseline patient and lesion characteristics.

**Patient characteristics (n = 25)**	
Age, years	59 ± 9
Male, n (%)	21 (84%)
Current smoker, n (%)	4 (16%)
Hypertension, n (%)	14 (56%)
Hyperlipidemia, n (%)	19 (76%)
Diabetes mellitus, n (%)	8 (32%)
Previous myocardial infarction, n (%)	0 (0%)
Body mass index, kg/m^2^	26 ± 4
Systolic pressure, mmHg	127 ± 14
Diastolic pressure, mmHg	74 ± 11
Mean blood pressure, mmHg	92 ± 10
Left ventricular mass, g	114 ± 32
Agatston score	265 (84, 461)
**Lesion characteristics of interested vessels (n = 30)**	
Focal lesion, n (%)	12 (40%)
Bifurcation lesion, n (%)	10 (33%)
Tandem lesion, n (%)	5 (17%)
Ostial lesion, n (%)	3 (10%)
**Stent characteristics (n = 36)**	
Stent length, mm	26 ± 11
Stent diameter, mm	2.96 ± 0.50

Data are presented as mean ± SD, median (interquartile range), or n (%).

**TABLE 2:** Baseline lesion and implanted stent characteristics.

Patient ID	Vessel	Lesion type	*L* (mm)	*D* (mm)	DS (%)	Stent type
P1	LAD	Bifurcation	28	3.5	60	Boston Scientific Synergy
P2	RCA	Tandem	14	4	60	Biosensors Biomatrix Neoflex
			28	4	95	Biosensors Biomatrix Neoflex
			32	3	80	Boston Scientific Synergy DES
P3	LAD	Focal	28	3	90	Neich Combo SDS
	LCX	Focal	31	2.25	75	Alvimedica Cre8
P4	LAD	Focal	18	2.5	80	Neich Combo SDS
P5	LAD	Focal	16	3	70	Boston Scientific Synergy DES
P6	LAD	Bifurcation	46	3	80	Alvimedica Cre8
	D2	Bifurcation	15	2.5	85	POBA with Kaneka Ikazuchi Zero Balloon
	LCX	Focal	46	2.5	95	Alvimedica Cre8
P7	LAD	Bifurcation	20	3	60	Alvimedica Cre8
P8	LAD	Focal	20	3	75	Alvimedica Cre8
P9	LAD	Focal	15	3.5	50	Abbott Vascular Xience Alpine
P10	LAD	Focal	19	3	60	MERIL Biomime DES
P11	RCA	Bifurcation	23	3.5	70	DESyne X2 (Elixir medical corporation)
P12	LAD	Bifurcation	48	2.5	81	Abbott Xience Xpedition DES
P13	RCA	Ostial	14	3.5	90	BIoFreedom DES
P14	LAD	Tandem	38	2.5	90	Bonston Scientific Synergy DES
			12	3.5	75	Bonston Scientific Synergy DES
P15	LAD	Focal	38	3	93	Bonston Scientific Synergy DES
P16	LAD	Bifurcation	24	2.5	72	Bonston Scientific Synergy DES
P17	LAD	Bifurcation	23	3.5	75	Xience Sierra DES
P18	LAD	Tandem	30	2.75	83	Medtronic Resolute ONYX DES
			30	2.25	75	Medtronic Resolute ONYX DES
P19	LCX	Ostial	20	3	80	Boston Scientific Agent DES
	LAD	Tandem	20	3	70	Bonston Scientific Synergy II DES
			20	2.5	80	Bonston Scientific Synergy II DES
P20	LAD	Focal	38	3.5	80	Orbus COMBO DES
	LCX	Focal	12	2.25	90	Bonston Scientific Synergy II DES
P21	LAD	Ostial	28	3	70	Abbott Xience Sierra DES
P22	LAD	Bifurcation	18	2	80	Abbott Xience ALPINE DES
P23	RCA	Tandem	48	3	77	Bonston Scientific Synergy DES
			28	3	77	Bonston Scientific Synergy DES
P24	LAD	Focal	38	2.5	70	Synergy DES
P25	LAD	Bifurcation	18	3.5	81	Abbott Xience Sierra DES

DS: Diameter stenosis; *L*: Stent length; *D*: Stent diameter. LAD: left anterior descending; LCX: left circumflex; RCA: right coronary artery.

### Comparison of FFR_B_ and Invasive FFR Before Stenting

**FIGURE 4:**
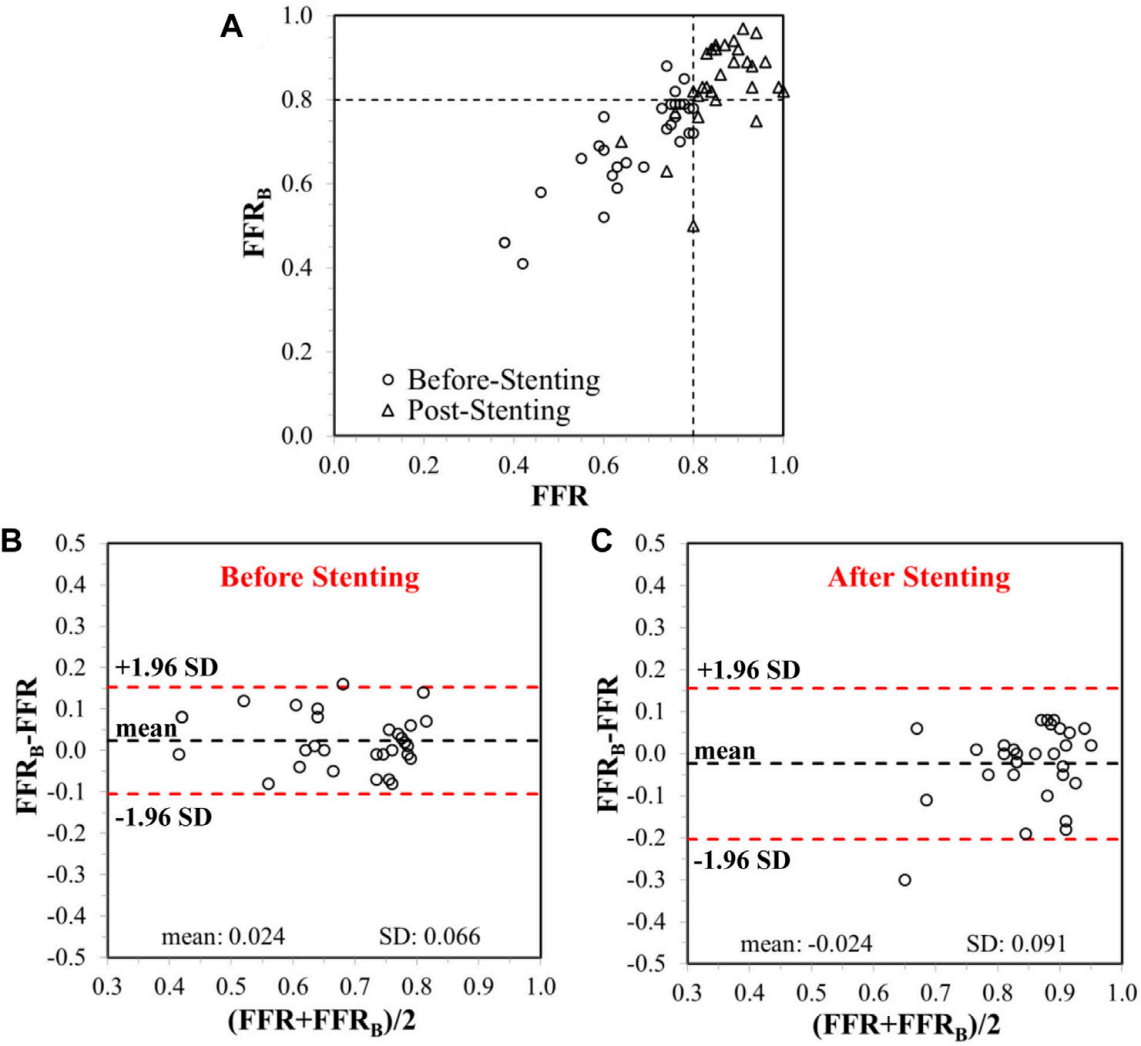
**(A)** Correlation between invasive FFR and FFR_B_, before and after stenting. The vertical and horizontal dashed lines represent the thresholds for FFR and FFR_B_, respectively, for ischemic classification of lesions. Bland-Altman plots showing limits of agreement on differences between FFR and FFR_B_ **(B)** before and **(C)** after stenting.

CFD simulations were successfully conducted in all patients. Each simulation took 0.4–1.5 h with a Dell T7800 workstation. Before stenting, mean FFR_B_ was 0.69 ± 0.12 and mean FFR was 0.67 ± 0.13. The mean difference (bias) between FFR_B_ and FFR was 0.024 [95% limit of agreement (LoA): −0.11, 0.15]. A comparison of FFR_B_ and FFR before stenting is presented in Figure 4A and shows excellent correlation of 0.86 (*p* < 0.001) between FFR_B_ and FFR. The corresponding Bland-Altman plot is shown in Figure 4B.

### Comparison of FFR_B_ and Invasive FFR After Stenting

**FIGURE 5:**
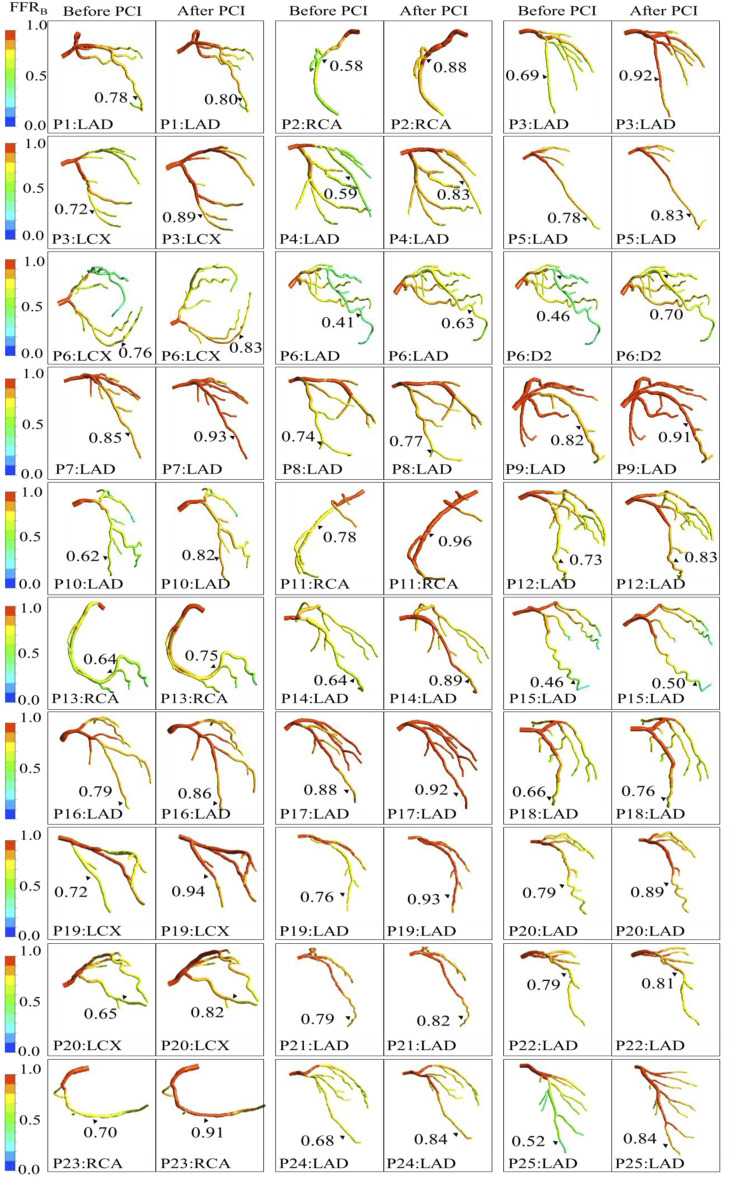
Comparison of computationally simulated FFR (FFR_B_) before and after stenting for all 25 patients (30 vessels).

After stenting, mean FFR_B_ was 0.84 ± 0.10 and mean FFR was 0.86 ± 0.08. The mean difference (bias) between FFR_B_ and FFR was −0.024 (95% LoA: −0.21, 0.16). A comparison of FFR_B_ and FFR after stenting is presented in Figure 4A demonstrating fair correlation (*r* = 0.50, *p* < 0.001) between FFR_B_ and FFR. The corresponding Bland-Altman plot is shown in Figure 4C. Diagnostic accuracy, sensitivity, specificity, positive predictive value (PPV) and negative predictive value (NPV) of FFR_B_ for predicting residual ischemia after stenting was 87, 80, 88, 57 and 96%, respectively. Figure 5 presents simulation results for individual patients and vessels before and after stenting with the corresponding FFR_B_ values.

### Reproducibility

For first observer, the median and interquartile range of derived FFR_B_ for replicated analyses of the selected 10 models were 0.83 (0.80, 0.89) and 0.82 (0.80, 0.90), respectively. The mean difference was 0.0018 with 95% LoA of (−0.017, 0.021). Intra-observer ICC and COV was 0.997 (95% CI: 0.988, 0.999) and 0.83% (95% CI: 0.43%, 1.22%), respectively.

For the second observer, the median and interquartile range of derived FFR_B_ was 0.81 (0.80, 0.87) for the same selected 10 models. Compared with the first set of analysis results by the first observer, FFR_B_ had a mean difference of 0.015 with 95% LoA of (−0.035, 0.064). Inter-observer ICC and COV were 0.976 (95% CI: 0.915, 0.994) and 2.34% (95% CI: 1.22%, 3.48%), respectively.

## Discussion

In our study, we developed a rapid method for computing FFR_B_ before and after stenting and compared it with invasive FFR. FFR_B_ increased significantly from 0.69 ± 0.12 before stenting to 0.84 ± 0.10 after stenting. The Pearson correlation coefficients between FFR_B_ and FFR were 0.86 before and 0.50 after virtual stenting. FFR_B_ demonstrated 87% accuracy for identifying post-stenting ischemic lesions.

A similar approach was adopted by Kim et al. (Kim et al., 2014) to evaluate residual ischemia post PCI *via* non-invasive FFR_CT_. They reported correlation coefficients between FFR_CT._ and invasive FFR of 0.60 and 0.55 before and after stenting, respectively, while the coefficients in our study were 0.86 and 0.50. When classifying FFR >0.80 post stenting as ischemia free, the accuracy, sensitivity, specificity, PPV and NPV for diagnosing residual ischemia in their study was 96, 100, 96, 50, and 100% while our corresponding values were 87, 80, 88, 57, and 96%, respectively. The lower PPV in these studies can be attributed to the relatively few residual ischemia cases after intervention (e.g., in the present study we only have five of 30 vessels with residual ischemia). In another study, Bom et al. (Bom et al., 2021) investigated the accuracy of FFR_CT_ planner to predict the hemodynamic gain of PCI. Results have shown that the FFR_CT_ planner tool demonstrated significant agreement with invasive post-PCI FFR values (*r* = 0.53 pre-PCI and 0.41 post-PCI, both *p* < 0.001) and with changes in FFR values after PCI (*r* = 0.57, *p* < 0.001).

Gosling et al. (Gosling et al., 2019) used ICA as the imaging modality for simulating stent placement. Using ICA, they obtained improved correlations between noninvasive FFR and invasive FFR before and after stenting reporting correlations of 0.87 and 0.80, respectively. Their method, however, relied on ICA images. To achieve real time prognosis similar to or shorter than the time required for FFR measurements, simplifying the computations *via* implementation of generic distal boundary conditions and/or limiting the vessels for CFD analysis (without considering side branches) can be explored and accuracy would need to be verified.

Our method relies on CTCA imaging and employs complete coronary artery tree reconstruction with patient-specific distal boundary conditions. Although Kim et al. (Kim et al., 2014) also used CTCA images, their computationally simulated FFR values were based on transient simulations of the entire cardiac cycle and consequently were computationally demanding. In comparison, our method relies on steady state simulation and combines a novel iterative algorithm to implement patient-specific outlet boundary conditions which considerably lowers the computation time (Zhang et al., 2016). We anticipated that this would lead to shorter turnaround time, which is an important consideration in the proposed application for decision-making in the clinical setting. Compared with the transient simulation employed, our methodology involves steady state simulation along with novel resistance boundary conditions that achieved equivalent accuracy in reference to invasive FFR at a computational time 1/16 of that required for full transient simulation (Zhang J.-M. et al., 2015). Of note, we have applied this reduced order CFD method to model both pre- and post-virtual stenting coronary hemodynamics, which constitute an original application.

As shown in the case example of treatment planning for tandem lesions (see Supplementary Material and Supplementary Figure 5), using non-invasive FFR prediction, we can determine the importance of each lesion’s role in the hemodynamics of tandem lesions and decide the optimal treatment strategy for tandem lesions. All of these results corroborate the potential of our technology as a tool for assisting treatment planning in the catheterization laboratory. Our technology allows operators latitude and flexibility to explore various stenting strategies virtually on a per-patient basis, before the invasive procedure, and depending on the hemodynamic effects of various options, choose the optimal strategy. In that manner the hemodynamic effects of the different strategies can be assessed by FFR_B_.

The current study simulated post-stenting hemodynamics on the CTCA model by incorporating information on implanted stent characteristics. This included actual stent lengths and diameters as chosen by the interventionists (see Table 2), and stenting locations referred from invasive angiography. This is for the purpose of validation against actual hemodynamic parameters that were measured after coronary intervention, so as to demonstrate the proof-of-concept of using our simulation models to predict the hemodynamic outcomes of coronary stenting.

For the proposed use of simulated models for treatment planning, the hemodynamic results of both stenting strategy (choice of which discrete lesions to stent or not to stent) and stent selection (stent length and diameter) can be simulated. With regards to the latter, stent characteristics can be determined from the luminal geometry on CTCA. For example, we can generate a curve to represent coronary lumen area changes along the centerline of the stenosed coronary artery from 3D curved multiplanar reformatted CTCA. A finite length “S” represents the stenotic site with minimum lumen area. The proximal (“P”) and distal (“D”) ends of the stenotic site correspond to the proximate and distal points on plotted curves with maximal absolute values of change of slope on either side of “S”, respectively. The distance between points “P” and “D” can be used to determine the stent length, while the average of the diameters corresponding to points “P” and “D” respectively can be used to estimate the stent diameter.

Hence, the present technology has the dual capability of diagnosing as well as planning and predicting therapeutic benefits of a revascularization strategy for CAD patients. The clinical application of this tool can thus reduce unnecessary interventions, procedural time, radiation dose and costs.

Our study had limitations. The number of patients enrolled was relatively small (25 patients with FFR measurement on 30 vessels) in a predominantly Asian population. A larger cohort size will be needed to corroborate these initial findings. Thus, we propose these findings be considered as a “proof-of-concept”. Another clinical trial on a much larger cohort of patients undergoing PCI to further validate the current methodologies is prerequisite before applying this tool in routine clinical practice. Second, this technology relies on CTCA imaging for reconstructing patient-specific anatomy and currently is only applicable in patients with diagnostic quality CTCA images. Third, the patient-specific geometry is based on the CTCA images taken before revascularization and hence do not consider anatomical changes (e.g., local curvature changes due to vessel compliance with stent) or microvascular injury introduced during the PCI procedure. Fourth, although in a previous study our non-invasive FFR_B_ technology was shown to have good diagnostic performance in classifying ischemia causing lesions (Zhang et al., 2016), the results were based on retrospective data. The complete data of our current prospective study on Asian cohort will be released to validate the clinical utility of the proposed methodology. Fifth, the present method does not consider different stent designs, material characteristics or drug coatings. These factors are more likely to affect long term stent performance than the acute hemodynamic improvement that is studied here. Sixth, hyperemia was induced by either intravenous infusion or intracoronary bolus of adenosine, nonetheless, prior studies have reported that intravenous infusion of adenosine yielded identical FFR result compared with intracoronary bolus (Schlundt et al., 2015). Lastly, the inlet static pressure was set at 6.8 mmHg below the patient-specific mean blood pressure (MBP) to account for the hyperemia effect of adenosine (Wilson et al., 1990). After stenting, even though the stenotic luminal area is enlarged (e.g. diameter stenosis decreased), this assumption is still valid since the adenosine effect on MBP will still dominate. While we cannot measure actual MBP post-virtual stenting, the effects of any potential effects on FFR_B_ are unlikely to be clinically significant. In a prior study, we estimated a FFR_B_ change 0.01 for every 6.25 mmHg unit change of MBP (Zhang et al., 2020).

## Conclusion

Computationally simulated FFR is highly reproducible, readily obtainable from standard CTCA images with reduced-order CFD and novel boundary conditions. This finding is promising for noninvasive detection of hemodynamically significant coronary stenosis and holds potential as a virtual coronary stent implantation planning tool to predict the hemodynamic effects of stenting in CAD patients.

## Data Availability

The original contributions presented in the study are included in the article/Supplementary Material, further inquiries can be directed to the corresponding author.

## Abbreviations

CAD, coronary artery disease; CFD, computational fluid dynamics; COV, coefficient of variation; CTCA, computed tomography coronary angiography; DBP, diastolic blood pressure; FFR, fractional flow reserve; ICA, invasive coronary angiography; ICC, intra-class correlation; LAD, left anterior descending artery; LoA, limits of agreement; MBP, mean blood pressure; NPV, negative predictive value; PCI, percutaneous coronary intervention; PPV, positive predictive value; SBP, systolic blood pressure; SD, standard deviation.
