# Supplementary material for: Computed Tomography Coronary Angiography and Computational Fluid Dynamics Based Fractional Flow Reserve Before and After Percutaneous Coronary Intervention
Source: Front Bioeng Biotechnol. 2021 Sep 7;9:739667. doi: 10.3389/fbioe.2021.739667 (PMC8452917; doi:10.3389/fbioe.2021.739667)
Supplement: Supplementary file 1 [file DataSheet1.DOCX]

Supplementary Material

**Resistance Boundary Condition and User Defined Function for Simulation**

We have used resistance boundary condition for the outlets. Specifically, a user defined function (UDF) was compiled to provide the pressure at each coronary outlet as

$P_{i}=P_{0}+R_{i}Q_{i}$ (1)

Here *P_i_* and *P*_0_ represent the pressure at the *i*-th outlet and its reference pressure respectively. *Q_i_* and *R_i_* represent the flow rate and the downstream vasculature resistance of the *i*-th outlet respectively. The former can be obtained through integration of the velocity at the *i*-th outlet surface.

An under-relaxation scheme was used to update *P*_0_ and *R_i_* to ensure smooth convergence during iterations after initializing them as 20 mmHg and 100,000,000 Pa·s/m^3^, respectively,

$P_{0,new}=\left(1-\alpha\right)P_{0,old}+\alpha\left(P_{inlet}-\sum_{i=1}^{N} \left(\frac{P_{i}-P_{0}}{N_{i}}\right)\right)$ (2)

$R_{i,hypermia,new}=\left( 1-\alpha\right)R_{i,hypermia,old}+\alpha(KN_{i}\frac{P_{\mathrm{inlet}}-P_{0}}{Q_{\mathrm{inlet}}})$ $R_{i,new}=\left( 1-\alpha\right)R_{i,old}+\alpha\left( KN_{i}\frac{P_{inlet}-P_{0}}{Q_{inlet}} \right)$ (3)

Here, *P*_0_*_,old_* and *P_0,new_* represented the reference pressure in the last and next iteration respectively. *R_i,old_* and *R_i,new_* represented the resistance values in the last and next iteration respectively with *α* being the under-relaxation factor.

*P_inlet_* represents the patient-specific aortic pressure, which can be estimated from the branchial artery pressure. Myocardial mass measured from CTCA images, can be used to estimate the total coronary flow at resting, *Q_inlet_* (Hamada et al., 1998, Wieneke et al., 2005).

*N_i_* represents the ratio of the downstream vasculature resistance of the *i*-th outlet to that of the coronary artery tree, which can be estimated from the scaling law (Zhou et al., 1999). *K* represents the reduction of coronary resistance under hyperemia, which was assumed to be 0.21 here, according to the measurement of the response to adenosine by Wilson et al. (Wilson et al. 1990).

During the CFD iterations, *P_0_* and *R_i_* were automatically updated until the total outflow from all the outlets matched the inflow rate at hyperemia.

**Case Example of Treatment Planning for Tandem Lesions**

To explore this technology in treatment planning for complex disease, **Supplementary Fig. 5** shows a case study of an elderly man with tandem lesions. Tandem lesions are interesting because they render invasive FFR inefficacious upon determining functional significance of an individual lesion when the other lesions have not been treated or removed (Pijls et al., 2000; De Bruyne et al., 2000). As a result, important physiological data is not available to the interventionist who then has to make treatment decisions based upon clinical experience. Using virtual stenting, this impediment is easily overcome by selectively removing one of the lesions in the computational model.

For this patient, an invasive FFR of 0.63 in the distal LAD confirmed the functional significance of the tandem lesion. The revascularization of both lesions resulted in an FFR of 0.92 after intervention. We predicted the FFR non-invasively for each lesion, as shown in **Supplementary Fig. 5 (e)** and **(f)**. These results show that the proximal lesion is hemodynamically significant whereas the distal lesion is not hemodynamically significant. In this scenario a revascularization strategy focusing only on the proximal lesion would have ensured ischemia free conditions.

De Bruyne, B., Pijls, N.H., Heyndrickx, G.R., Hodeige, D., Kirkeeide, R., and Gould, K.L. (2000). Pressure-derived fractional flow reserve to assess serial epicardial stenoses: theoretical basis and animal validation. Circulation. 101, 1840–1847. doi: 10.1161/01.cir.101.15.1840

Hamada, M., Kuwahara, T., Shigematsu, Y., Kodama, K., Hara, Y., Hashida, H., et al. (1998) Relation between coronary blood flow and left ventricular mass in hypertension: noninvasive quantification of coronary blood flow by thallium-201 myocardial scintigraphy. Hypertens. Res. 21, 227–234. doi: 10.1291/hypres.21.227

Pijls, N.H., De Bruyne, B., Bech, G.J., Liistro, F., Heyndrickx, G.R., Bonnier, H.J., et al. (2000). Coronary pressure measurement to assess the hemodynamic significance of serial stenoses within one coronary artery: Validation in humans. Circulation. 102, 2371–2377. doi: 10.1161/01.cir.102.19.2371

Wieneke, H., von Birgelen, C., Haude, M., Eggebrecht, H., Möhlenkamp, S., Schmermund, A., et al. (2005). Determinants of coronary blood flow in humans: quantification by intracoronary Doppler and ultrasound. J. Appl. Physiol. 2005. 98, 1076–1082. doi: 10.1152/japplphysiol.00724.2004

Wilson, R.F., Wyche, K., Christensen, B.V., Zimmer, S., and Laxson, D.D. (1990). Effects of adenosine on human coronary arterial circulation. Circulation. 82, 1595–1606. doi: 10.1161/01.cir.82.5.1595

Zhou, Y., Kassab, G.S., and Molloi, S. (1999). On the design of the coronary arterial tree: a generalization of Murray’s law. Phys. Med. Biol. 44, 2929–2945. doi:10.1088/0031-9155/44/12/306

**Supplementary Table 1. CT Scanners Employed in the Study along with their Specifications**

| **Scanner** | **X-ray Source, (n)** | **Slices/ Detector rows (n)** | **Spatial resolution(mm)** | **Gantry rotation time (ms)** | **Temporal resolution(s)** |
| --- | --- | --- | --- | --- | --- |
| Toshiba Aquilion ONE | 1 | 320 | 0.50 | 350.00 | 0.175 |
| Canon Aquilion ONE Genesis 640 | 1 | 640 | 0.22 | 350.00 | 0.175 |
| Philips Brilliance iCT | 1 | 256 | 0.42 | 270.00 | 0.135 |
| Siemens Somatom Force | 2  (95^0^ apart) | 384 | 0.24 | 250.00 | 0.066 |


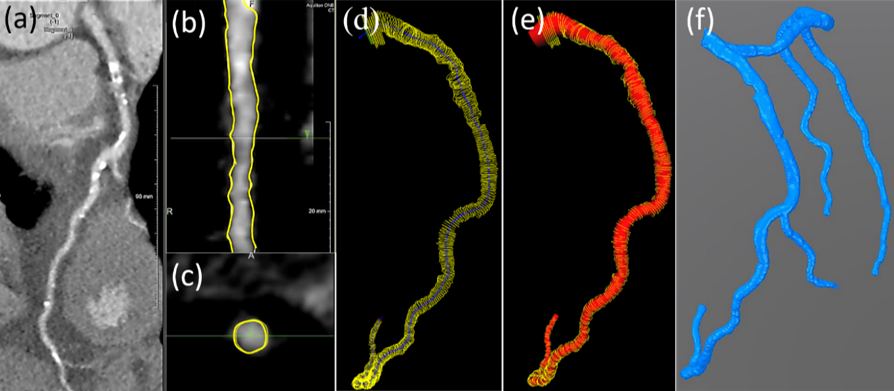


**Supplementary Figure 1.** **Details of segmentation and 3D model reconstruction**: (a) Multi planar-reformatted image of left anterior descending artery (LAD), (b) automated contour detection using QAngio CT (Research Edition v3.0.37.0, Medis, Leiden, The Netherlands) in the longitudinal view, (c) transverse views, (d) centerline of the vessel along with the contours delineating the lumen, (e) reconstructed LAD using 3D workbench, and (f) full reconstructed 3D model.


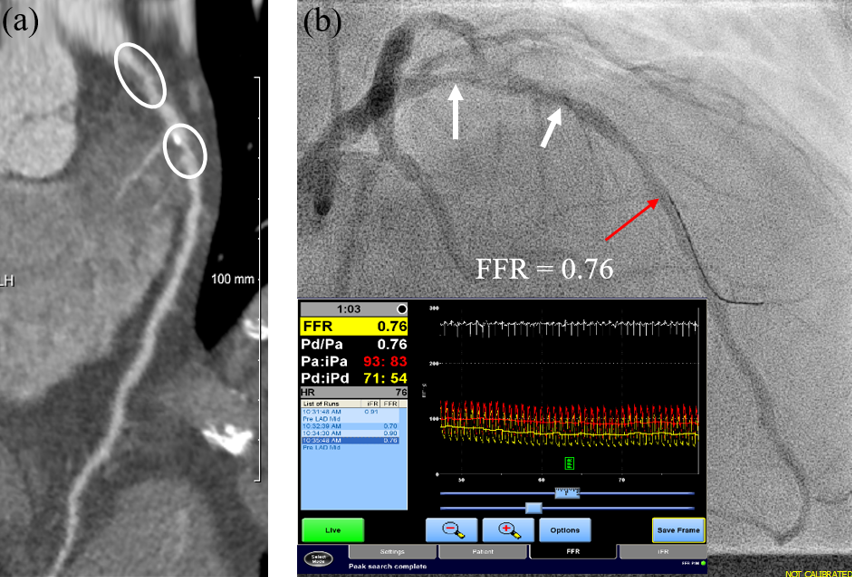


**Supplementary Figure 2.** **Example case of CTCA and FFR measurement in LAD for a 61 years old Chinese male**: (a) Multi-planar reconstruction of CTCA images showing the lesions in the proximal and mid LAD, (b) Corresponding ICA image with white arrows indicating stenosis along the LAD and measured FFR of 0.76 at the location indicated by the red arrow.


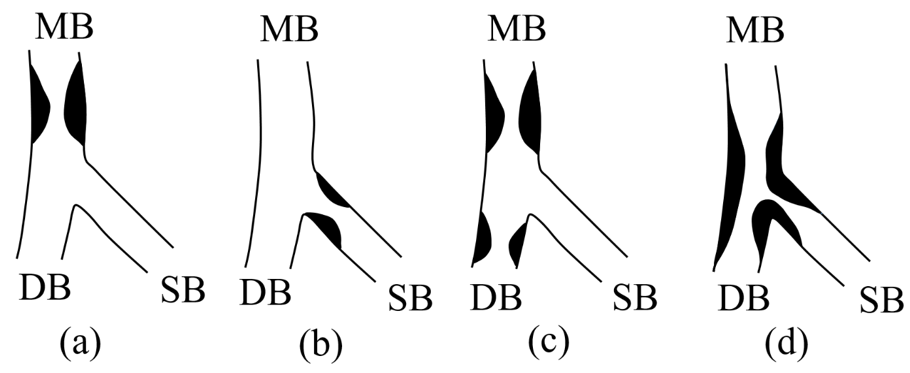


**Supplementary Figure 3. Classification of lesion types in the study**: (a) Focal Lesion, (b) Ostial Lesion, (c) Tandem Lesion, and (d) Bifurcation Lesion. Here, MB is Main Branch, DB is Daughter Branch and SB is Side Branch.

**Supplementary Figure 4. Histogram of invasive FFR values measured pre- (blue) and post-PCI (red).**


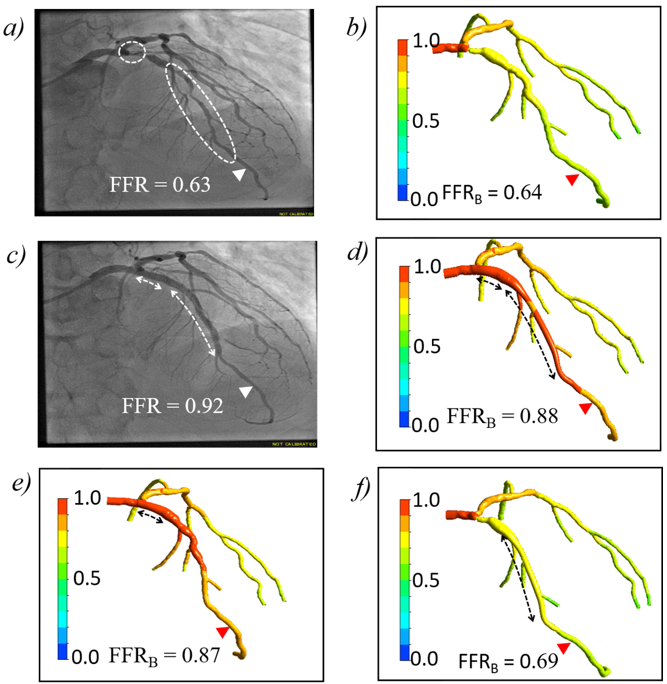


**Supplementary Figure 5. Application of virtual stenting for estimating true FFR in tandem lesions**. (a) ICA image of the patient highlighting the tandem lesions with invasive FFR measurement confirming the hemodynamic significance of the lesion. (b) FFR_B_ is in good agreement with invasive FFR, classifying the lesions as hemodynamically significant. (c) ICA image of the patient after intervention showing the two implanted stents with invasive FFR measurement confirming ischemia free conditions. (d) FFR_B_ obtained after virtual stent placement remains in good agreement with the corresponding invasive FFR measurement. (e) Stenting strategy 1: virtual stent placement in the proximal lesion showing the distal lesion to be non-hemodynamically significant. (f) Stenting strategy 2: virtual stent placement in the distal lesion showing the proximal lesion to be hemodynamically significant.
